# Morphological Variability of the Sural Nerve and Its Clinical Significance

**DOI:** 10.3390/jcm13206055

**Published:** 2024-10-11

**Authors:** Weronika Marcinkowska, Nicol Zielinska, Bartłomiej Szewczyk, Piotr Łabętowicz, Mariola Głowacka, Łukasz Olewnik

**Affiliations:** 1 Department of Anatomical Dissection and Donation, Medical University of Lodz, 90-419 Łódź, Poland; w.marcinkowska88@gmail.com; 2 Department of Clinical Anatomy, Masovian Academy in Płock, 09-402 Płock, Poland; nicol.zielinska@stud.umed.lodz.pl (N.Z.); bartlomiej.szewczyk@umed.lodz.pl (B.S.); piotr.labetowicz@umed.lodz.pl (P.Ł.); 3 Nursing Department, Masovian Academy in Płock, 09-402 Płock, Poland; m.glowacka@mazowiecka.edu.pl

**Keywords:** sural nerve, anatomical variations, sural biopsy, peripheral neuropathy, sural nerve graft, sural mononeuropathy, sural nerve entrapment

## Abstract

The sural nerve provides sensory innervation to the skin on the distal posterolateral third of the lower extremity. The morphological variants are characterized by high variability. However, it most commonly arises from a union of the medial sural cutaneous nerve and the peroneal communicating branch of the common fibular nerve. This article overviews the anatomical and clinical significance of the sural nerve. Despite the remarkable development of genetic diagnostics, sural nerve biopsy is still a very important tool to diagnose peripheral neuropathies such as diabetic, vascular and inflammatory neuropathies. Furthermore, the sural nerve is also commonly transplanted due to its characteristics. Such a procedure is applicable in cases of segmental nerve loss, but it is also used to restore potency in patients after radical prostatectomy. The knowledge of anatomical variants of the sural nerve is also crucial as it allows to minimize its damage during surgical procedures. Furthermore, during an ankle surgery, a nerve block can be used to complement anesthesia. The major aim of this work is to review contributions of the sural nerve to physiological and pathophysiological processes.

## 1. Introduction

**Figure 1:**
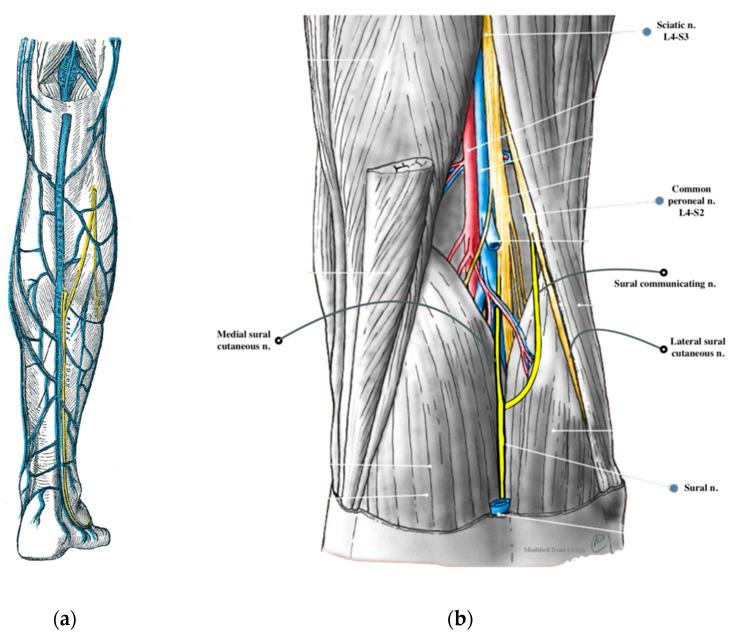
(**a**) The small saphenous vein and the sural nerve; Henry Vandyke Carter, public domain, via Wikimedia Commons [17]; and (**b**) the most common formation of the sural nerve; Robert Steele MS DO, via Wikimedia Commons [18].

The sural nerve (SN) is a sensory nerve of the lower limb that originates mainly from S1 and S2 nerve roots. The peripheral nerves that include the SN are supplied by vessels originating intrinsically from the vasa nervorum, which form a longitudinal network of small vessels connected by multiple anastomoses in the epineurium, perineurium and endoneurium, and extrinsically from parallel arteries [1,2]. The SN is nourished proximally by a single superficial sural artery, while distally, it receives multiple supplies from the musculocutaneous and fasciocutaneous perforators of the posterior tibial and fibular arteries [3]. The SN commonly arises from the union of two major nerve branches, including the medial sural cutaneous nerve (MSCN), a branch of the tibial nerve (TN) and the peroneal communicating branch (PCB) of the common fibular nerve (CFN) [4,5,6,7,8]. However, it can also be a direct continuation of the MSCN or a combination of the MSCN and the lateral sural cutaneous nerve (LSCN), as a branch of the CFN [6,8,9,10]. The SN usually arises in the distal third of the calf and passes between the two heads of the gastrocnemius muscle lateral to the short saphenous vein (SSV) [1]. It then continues along the lateral border of the calcaneus tendon and wraps behind the lateral malleolus, where the SN gives off lateral calcaneal branches that terminate at the lateral aspect of the heel. The SN continues to the fifth toe, where it passes into the lateral dorsal cutaneous nerve (LDCN) (Figure 1) [10,11,12,13].

The main function of the SN is to provide sensory supply to the skin of the posterolateral lower third of the leg down to the lateral malleolus and the lateral aspect of the dorsum of the foot to the fifth digit [1,14].

Comprehensive studies on the SN consistently reveal its great variability of the course, as well as its distribution and formation. The formation from a combination of TN and CFN branches is most commonly described [4,5]. Moreover, some of the literature reports also confirm variants in which the MSCN and the LSCN run in parallel, and the SN is formed from a combination of the LSCN and the PCB [8].

The SN is also implicated in pathological conditions, as it plays a role in the diagnosis of peripheral neuropathies [15]. In addition, due to its superficial location, it is vulnerable to trauma, which can cause entrapment of the SN presenting a sensory dysfunction within the areas supplied by the nerve [16].

The aim of this review article is to summarize the current knowledge of the morphological and clinical significance of the SN. An understanding of its anatomical variants is useful for procedures such as biopsy and nerve grafting. In addition, detailed knowledge is crucial to carry out effective and safe surgical procedures at the site of the SN and avoid its iatrogenic injury.

## 2. Anatomical Variations of Sural Nerve

Various types of origins and sites of SN formation were thoroughly described in recent studies. Each author’s research work varies in the number of cases studied and the method of identification of the location of the course of the SN. Huelke examined 352 lower extremities of American cadavers [4]. The SN was localized by isolating the TN and CFN in the popliteal fossa, which were then dissected to reveal the origin of the MSCN, LSCN and PCB [4]. Mahakkanukrauh and Chomsung studied 152 limbs of Thai cadavers. Through a longitudinal skin incision, parallel with the posterior border of the lateral malleolus, the SN was located. Then, the course of the SN was observed at the site where it splits into the MSCN and LSCN [9]. However, Ugrenovic et al. performed their examination on 200 fetal lower extremities. A dissection was performed through two horizontal incisions at the level of the gluteal sulcus and between the medial and lateral malleolus, which were united by one longitudinal cut [5]. Pyun and Kwon conducted their study on 26 lower limbs. The investigation not only involved a determination of the type and frequency of anatomical variation but also a relationship between the anatomical variation and nerve conduction study(NCS), which is useful in the diagnosis of generalized peripheral neuropathy or focal neuropathy in lower extremities [10]. Shankar et al. dissected 102 limbs of embalmed fetuses of Indian origin, as described by Ugrenovic et al. [5,6]. Kavyashree et al. included in their study 50 lower limbs of cadavers, in which two horizontal incisions were made at the junction of the medial and lower 1/3 of the thigh and at the lower end of the lateral malleolus, which were connected by a vertical incision [7]. However, Steele et al. used 208 limbs of American cadavers, in which the location of the SN was determined by incising the skin between the posterior border of the lateral malleolus and the tendon of the calcaneus [8].

Based on the anatomical variants of the SN described in the literature, Ramakrishnan et al. distinguished six types of its formation [11]. Type 1 is subdivided into type 1A, in which the SN is a union of the MSCN arising from the TN and PCB originating from the CFN, and type 1B, in which the SN is formed by a combination of the MSCN of the TN and PCB emerging directly from the LSCN of the CFN. In type 2, the SN is created by a union of the MSCN and the LSCN originating from the TN and CFN, respectively. Type 3 has been subdivided into type 3A, in which the SN is a continuation of the MSCN with the absence of the PCB and the LSCN, and type 3B, in which the SN is a continuation of the MSCN with the absence of the PCN, but with the LSCN, which is independently present. In type 4, the SN is formed only by the PCB, and in type 5, by the LSCN only with independent or absent MSCN. However, in type 6, the SN is produced directly from the sciatic nerve (SCN) [11]. Shankar et al. also described such a variant [6]. In addition, Steele et al. [8] identified two anatomical variants, i.e., type 7 and type 8, which were not defined by the Ramakrishnan et al. criteria [8,11]. Both variants are characterized by an independent parallel course of the MSCN and LSCN. In type 7, the SN is formed by the union of the PCB with the parallel LSCN, while in type 8, the SN is a portion of the LSCN that penetrates the deep and superficial fascia in the sura [8].

Table 1 summarizes the frequency of each type of SN formation according to previous investigation trials. For most authors, the prevalent type was type 1 [4,5,6,7,8]. Type 2, being the predominant type, was noted only by Mahakkanukrauh and Chomsung [9]. However, Pyun and Kwon did not find type 1 in their study [10]. Among the American population, the most common formation of SN is type 1. However, in the Thai population, type 2 was the most commonly observed. In order to determine the site of SN formation, some authors divided the calf into four equal parts starting with the popliteal fossa and finishing with the lateral malleolus. The third quarter of the limb was the most common site of SN origin [4,5,8]. Nevertheless, the site of SN formation was variable. In the investigations performed by Mahakkanukrauh and Chomsung and Kavyashree et al., the SN was found more frequently in the distal part of the leg (Table 2) [7,9].

The SN is exposed to the risk of iatrogenic injury due to its anatomical variants and various sites of its formation. Moreover, due to its surgical and clinical implications, it is important to know the anatomy of the SN. These include its role in the diagnosis of peripheral neuropathies via biopsy. Furthermore, characteristic features of the SN make it a perfect donor in transplantations [11].

## 3. Diagnostics

Clinical manifestations of SN pathology are generally nonspecific [19]. SN injury may manifest as hypoesthesia, neuropathic pain and even affect the patient’s mental state and quality of life [20]. Therefore, imaging techniques are necessary to detect the nerve and assess its condition, facilitating early diagnosis and prompt treatment. The SN can be assessed using ultrasound with high-frequency transducers. Nevertheless, the small size of the nerve can be an obstacle to its adequate imaging. Moreover, the limitations in visualizing interfascicular connections and small fascicles within clusters require careful interpretation. However, lower frequency transducers are necessary when the nerve is compressed by the tumor [21,22].

The SN is best recognized about 10 cm above the calcaneus, where it attaches to the SSV and laterally to the Achilles tendon. The nerve divides at the level of the ankle joint into about three smaller branches, which are difficult to visualize under normal conditions. On ultrasound, a normal SN resembles a typical peripheral nerve [19]. Its internal structure is composed of hypoechoic nerve fascicles surrounded by hyperechoic connective tissue [21].

In SN pathologies, hypoechoic thickenings were the most common manifestations. In inflammation, the prolonged segment of the nerve is affected by edema, and ultrasound shows hypoechoic thickening of the involved segment of the nerve, with a loss of intraneural hypoechoic tissue circumferentially surrounded by interfascicular epineurium. Small fascicles may appear as a single undifferentiated structure and may be indistinguishable from the surrounding interfascicular epineurium or the cluster of fascicles on ultrasound. The differentiation of individual fascicles is facilitated by larger interfascicular distances, probably due to the amount of interfascicular epineurium [22,23]. However, compressive neuropathies result in the venous congestion and compression of the vasa nervorum, which leads to swelling and results in a fusiform hypoechoic thickening of the nerve. Additionally, color Doppler imaging is performed to evaluate the presence of local hypervascular lesions [19]. The SN located close to the SSV and Achilles tendon increases the risk of injury during vascular and orthopedic surgeries [24]. During an examination of pathological conditions, the local pressure exerted by a transducer can cause a burning pain in the neuroma, making it easier to detect its exact location [21].

Due to high anatomical variability, ultrasound imaging of the nerve is useful in preventing iatrogenic injury, especially during arthroscopic ankle surgery. In addition, US allows us to quickly and effectively localize the SN before anesthetizing the lower limb and facilitate harvesting the graft [25,26].

Magnetic resonance imaging (MRI) can also be used to explore the SN, although its fascicular anatomy remains difficult to visualize in a clinical setting. High-resolution high-field images provide a better visualization of the course of the SN in vivo to provide surgeons with prior knowledge of its location before surgery. Differences in the anatomy of the nerve observed on MRI enabled us to propose “safe zones” for surgical approaches, including the extensile lateral approach to the calcaneus, the sinus tarsi approach, the direct lateral approach to the lateral malleolus and the posterolateral approach to the ankle [27,28]. In addition, magnetic resonance microscopy revealed a more detailed fascicular image and a greater ability to visualize smaller fascicles compared to three-dimensional high-resolution ultrasound [22]. Imaging can obtain a basic characteristics of the nerve, while more detailed characteristics are not easily assessed in the case of a very small nerve [27,28].

The detailed structure of this small nerve can also be assessed using an optimized non-invasive micro-CT technique. For a detailed soft tissue evaluation, an X-ray contrast agent is required to adequately distinguish between the fascicles, the interfascicular epineurium, adipocytes and the surrounding air or layer holding the nerve. The micro-CT method provides high-resolution three-dimensional reconstruction of the entire scanned nerve and facilitates segmentation and tracking of the fascicles within the nerve [29].

## 4. Sural Nerve Biopsy

Peripheral neuropathies are frequently observed in clinical practice. Apart from being a non-invasive examination, SN biopsy is an important instrument to make a proper diagnosis. Besides, it can also be useful in revealing pathological mechanisms, especially in inflammatory neuropathies [15,30].

A nerve biopsy is not indicated if the cause of a neuropathy can be determined by clinical examinations and laboratory tests, as it is performed in diabetic, alcoholic and uremic neuropathies. Such patients are given a biopsy only to investigate the pathophysiology of the neuropathy. In contrast, suspected vasculitis and clinically significant peripheral neuropathy with no apparent cause are clear indications for nerve biopsy [31].

Due to its superficial course and anatomically facilitated location, the SN is the most commonly biopsied peripheral nerve. Moreover, it is a pure sensory and autonomic nerve that does not contribute to any loss of the motor function, and its sensory distribution is predictable and located on the dorsolateral side of the foot. Thus, permanent anesthesia in this region will not predispose the patient to ulcer formation [32].

During a biopsy, the patient is placed in the supine position. General anesthesia is induced, and a compression band is applied to the thigh. The skin and fascia are then incised at the lower pole of the gastrocnemius muscle, along the midline, about 10 cm below the popliteal fossa. Then, the SN location is identified. This nerve is commonly located medially under the lesser saphenous veins. A 2–3 cm section of the nerve is cut out using microsurgical techniques, and its proximal portion is implanted into the gastrocnemius muscle of the calf. The incisions are closed with sutures and a sterile dressing is applied [33].Two main components of peripheral nerves, axons and myelin are subject to histopathological evaluation. In addition, the examination also aims to detect diagnostic lesions such as amyloid deposits, sarcoid tubercles and vasculitis. Among other cells contained in a biopsy sample, endothelial cells and perecytes, which form the blood-nerve barrier, have a major role, especially in the diagnosis of inflammatory neuropathies [15].

SN biopsy plays a significant role in vascular neuropathy, a condition predominantly characterized by vasculitis and injury to blood vessels [34]. Over the course of the disease, an inflammatory process and fibrinoid necrosis damage the vasa nervorum, resulting in ischemia of the axon. The clinical presentation of neuropathy is varied and includes symmetric polyneuropathy as well as mononeuropathy [35]. Classical symptoms are predominant in lower extremities and include an acute pulsating pain in the onset, weakness and loss of sensation. Rarer manifestations are stocking-glove sensory neuropathies and pure motor neuropathies [36]. A suspicion of vasculitis is the most common indication for a nerve biopsy. Its histopathological results are aground for formulating a definitive diagnosis [37]. Pathological changes found in vasculitic neuropathy include fibrous necrosis with inflammatory infiltrates, asymmetric axonal loss, sub-endoneurial edema and perivascular microfasciculation [38].

Chronic inflammatory demyelinating polyneuropathy (CIDP) is the most common chronic immune-mediated polyneuropathy with a progressive or recurrent course lasting for more than 2 months [39,40]. The pathogenesis includes endoneurial inflammation and demyelination of the nerve, involving antibodies directed against antigenic components of the myelin sheath and complement [41]. CIDP is manifested with progressive proximal and distal weakness and loss of sensation in lower extremities and generalized areflexia. Sensory manifestations also include distal paresthesias, poor balance and impaired proprioception [41]. There are two clinical presentations of CIDP, i.e., typical and atypical. Typical CIPD is a symmetrical polyneuropathy that involves proximal and distal muscles. However, atypical CIPD includes distal acquired demyelinating symmetric, multifocal acquired demyelinating sensory and motor neuropathy as well as purely motor or sensory CIDP [39,40]. In CIDP, nerve biopsy is indicated in the event of uncommon clinical presentations, or if it is necessary to rule out nerve vasculitis or lesions caused by amyloid deposition [41]. Biopsy results are not homogeneous and comprise a loss of axons, inflammatory infiltrates, onion bulbs, fibers with a thin myelin sheath and segmental demyelination [38].

## 5. Diabetic Peripheral Neuropathy

Diabetic peripheral neuropathy (DPN) is a significant microvascular complication of and a risk factor of diabetic foot. It is the most common complication of both type 1 and type 2 diabetes and occurs in more than half of those affected. It is a cause of non-traumatic lower limb amputations and can lead to balance and gait impairment and neuropathic pain that is often unresponsive to treatment [42]. DPN is associated with increased mortality, so making an early diagnosis is important [43]. In patients with diabetes, regular foot examination plays an important role. During the examination, attention is drawn to the reduction or absence of all modalities of sensation, vibration or proprioception [44]. The most commonly damaged nerve in DPN is the SN, probably due to length-dependent exposure to chronic hyperglycemia and cardiovascular risk factors that induce metabolic and microvascular changes [45,46]. Distal symmetrical polyneuropathy is the most common type of DPN. First, it affects the longest axons and progresses gradually with the course of the disease, its severity and duration [45]. Early symptoms of the disease are often not noticeable until the disease is well advanced. Some patients experience numbness, paresthesia and neuropathic pain, which typically increases at night, causing insomnia. Additionally, there may be severe sensory loss in the presence of painful neuropathy symptoms, such as the so-called painful-less leg. The diagnosis of DPN is usually sufficient in a clinical context, without the need for specialized testing. However, atypical symptoms should prompt a referral for neurophysiological testing [44]. Currently, the gold standard for DPN diagnosis is nerve conduction studies. However, this examination provides limited information on the morphology of the nerves and surrounding structures. Due to the frequent involvement of the sural nerve, studies suggest that SN ultrasonography can be used as a diagnostic tool based on the cross-sectional area [46]. SN biopsy is a method used in the diagnosis of neuropathy. It allows for an evaluation of large myelinated and small unmyelinated nerve fibers, as well as blood vessels [47]. DPN is mostly characterized with a loss of myelinated fibers, which, along with axonal degeneration, is preceded by demyelination. Reduced myelin fiber density correlates with the severity of DPN [47].

## 6. Sural Mononeuropathy

Isolated SN neuropathy is a rare electrophysiological condition. The anatomical course and location of the nerve in the distal part of the lower limb and ankle make the SN prone to trauma, which is the most common etiology of neuropathy [48]. Neuropathy can be caused by injury, fracture and rupture within the ankle joint, and can also be manifested with chronic compression resulting from the presence of Baker’s cysts, tumors and intraneural ganglions [49,50,51,52]. Surgical procedures within the ankle joint and vein stripping procedures are also common etiologies [53]. Cases of neuropathy have also been reported in generalized inflammation and vasculitis [48,52].

The clinical presentation of mononeuropathy is dominated by pain, numbness, paresthesias and burning at the distribution regions of the SN. During sensory examination, impaired sensation was observed in most cases, while hyperesthesia or normal sensation were noted in the remaining cases [52].

Pain and loss of sensation in the ankle joint and lateral surface of the foot can occasionally suggest a misdiagnosis of S1 radiculopathy. In such a case, normal muscle strength and deep tendon reflexes, as well as reduced or absent sural sensory response amplitude observed upon electrophysiological examination, accompanied by normal other electrophysiological measurements and needle electromyography (EMG) examination, are helpful in the diagnosis [54].

## 7. Sural Nerve Entrapment

The SN can be easily prone to trauma and entrapment neuropathy because of its superficial anatomical location and close attachment to surrounding tissues [16]. The lateral aspect of the heel or foot are prevalent anatomical locations of SN compression [55]. The most common etiology of entrapment is superficial SN aponeurosis associated with a thickening of the fascia where the nerve passes superficially to the gastrocnemius muscle. Other causes of SN entrapment are repetitive ankle sprain, osperoneum fracture and fracture of the base of the fifth metatarsal bone, articular cystchronic Achilles tendonitis or space-occupying lesions, such as ganglion [55,56].

The clinical presentation of SN entrapment includes pain, burning, tenderness and abnormal sensation in the posterolateral region of the distal leg and lateral part of the foot to the fifth digit [1]. Similar symptoms can be observed in sacral sciatica, piriformis syndrome, exertional compartment syndrome and the popliteal artery entrapment. Thus, these conditions should be considered in a differential diagnosis. The diagnosis is mainly based on clinical findings, and the appearance of tenderness in the distal cutaneous distribution of the injured peripheral nerve elicited to percussion at the site of the proximal course of the nerve near the neck of the fibula (positive Hoffman-Tinel’s sign) can confirm the diagnosis, despite normal neurological findings. Because this condition can be induced by a trauma, a radiological examination is the first-line examination [55]. Treatment involves conservative management, including a removal of the offending agent or surgical removal of space-occupying masses and neurolysis, in cases of fascia-related compression [16].

## 8. Sural Nerve Graft

An SN graft is considered the gold standard in the reconstruction of an affected peripheral nerve. It enables regeneration of the end organ by providing a scaffold for regenerating axons and also promotes axonal regeneration through a delivery of Schwann cells [57]. The appropriate length and calibration of the SN, easy and quick harvesting and its straight course with minimal branching makes it a good donor nerve. In the classical open technique, which provides maximum exposure and minimal risk of injury, the nerve is harvested through a long incision from the distal to proximal side of the limb. In this technique, an unsightly scar can occur, which is its main disadvantage. Nerve harvesting can also be performed in a stepladder manner through a shorter incision using a tendon stripper, nerve stripper and also with the application of endoscopic techniques [58,59,60,61]. SN grafting improves the sensory and motor function. Yet, it does not entirely restore this function. In addition, the outcome depends on many factors, such as the location of the nerve interruption, duration of the procedure, the length of the nerve gap, but also the patient’s general condition and comorbidities [57,61].

SN grafts can be applied in order to treat segmental motor or sensory nerve loss and to lengthen the nerve in brachial plexus injuries [57]. Moreover, they are commonly used to restore potency during radical prostatectomy [14]. The graft procedure is also applied in corneal neurotization in patients suffering from neurotrophic keratitis to restore sensation in the cornea, which is performed by prolonging a functional sensory nerve and redirecting its axons toward the damaged cornea [57].

## 9. Surgical Implication

In clinical and surgical management, knowledge of anatomical variations and the course of the SN is crucial as it enables to prevent iatrogenic injury. The SN can be damaged by incisions of the posterior leg, lateral ankle or lateral foot region. Due to the fact that fractures in lower extremities are treated surgically, a malleolar fracture can be an additional complication factor [13,62]. A close relationship between the SSV and the SN can contribute to nerve damage during surgical stripping or thermal ablation of the SSV. Furthermore, the course of the nerve close to the lateral border of the calcaneal tendon increases the risk of nerve injury during surgical repair of the calcaneal tendon [14]. A precise knowledge of anatomical variants of the SN is also relevant to identify the localization of the nerve during local and regional anesthetic techniques [13]. Because of the superficial course of the SN, its blockade can be used as adjunct to general anesthesia in ankle surgery. The risks of this procedure include pain, bleeding, infection and allergic reaction to the anesthetic [1].

## 10. Conclusions

Knowledge of the detailed course and anatomical variants of the SN is essential for surgeons during procedures performed on the ankle joint. Clinicians should consider the variation in the course of the nerve when assessing potential sensory deficits and planning procedures that require anesthesia of the plantar surface of the foot. The most prevalent SN formation pattern was the union of the medial sural cutaneous nerve with the peroneal communicating branch. Moreover, the most common SN formation sites were the third quarter of the leg. The SN is significant in clinical practice. It is one of the most commonly involved nerves in diabetic neuropathy. In addition, the SN is the target of electrophysiological studies and nerve biopsies to diagnose peripheral neuropathies. The SN can also be used as a nerve graft in reconstructive surgery, especially in cases of peripheral nerve injury.

## Figures and Tables

**Table 1:** Frequency of SN formation observed by various authors.

	Number of Cases	Type 1 ^1^ (%)	Type 2 ^2^ (%)	Type 3 ^3^ (%)	Type 4 ^4^ (%)	Type 5 ^5^ (%)	Type 6 ^6^ (%)	Types 7 and 8 ^7^ (%)
Huelke (1957) [4]	352	80.7	-	19	0.3	-	-	NA
Mahakkanukrauh and Chomsung (2002) [9]	152	0.7	67.1	32.2	-	-	-	NA
Ugrenovic et al. (2005) [5]	200	58.5	9	26	-	1.5	-	5
Pyun and Kwon (2008) [10]	26	-	76.9	15.4	-	-	-	7.7
Shankar et al. (2010) [6]	102	29.4	-	26.5	22.5	-	13.7	7.8
Kavyashree et al. (2013) [7]	50	72	-	28	-	-	-	NA
Steele et al. (2021) [8]	208	41.4	8.7	34.6	0.5	0.5	-	14.4

^1^ The SN formed by the union of the MSCN of the TN and the PCB; ^2^ the SN formed by the union of the MSCN of the TN and the LSCN of the CFN; ^3^ the SN formed by the continuation of the MSCN with absent PCB and LSCN; ^4^ the SN formed by the PCB alone; ^5^ the SN formed by the LSCN alone with independent or absent MSCN; ^6^ the SN formed directly from the SCN; ^7^ and both variants are characterized by an independent parallel course of the MSCN and LSCN. In type 7, the SN is formed by the union of the PCB with the parallel LSCN, while in type 8, the SN is a portion of the LSCN that penetrates the deep and superficial fascia in the sura.

**Table 2:** Frequency of the site of SN formation.

	Upper Quarter of the Leg (%)	Second Quarter of the Leg (%)	Third Quarter of the Leg (%)	Fourth Quarter of the Leg (%)	In the Ankle Area (%)
Huelke (1957) [4]	69 (24.3)	48 (16.9)	104 (36.6)	63 (22.2)	-
Mahakkanukrauh and Chomsung (2002) [9]	6 (5.9)	-	2 (1.9)	69 (67.4)	26 (25.5)
Ugrenovic et al. (2005) [5]	2 (1.6)	35 (28.0)	81 (64.8)	7 (5.6)	-
Kavyashree et al. (2013) [7]	1 (2.8)	2 (5.6)	11 (33.3)	22 (58.3)	-
Steele et al. (2021) [8]	20 (9.6)	17 (8.2)	53 (25.5)	14 (6.7)	-

## Data Availability

The data used in this article are sourced from materials mentioned in the References section.
